# Beyond the Alpha: Extra‐Pair Paternities and Male Reproductive Success in a Primate Multilevel Society

**DOI:** 10.1002/ece3.71749

**Published:** 2025-07-07

**Authors:** Fan Wu, Jia Liu, Derek W. Dunn, Yixin Shang, Shiyu Jin, Huihui Du, Yuanchun Wu, Yiyi Men, Guoliang Chen, Gang He, Baoguo Li, Songtao Guo

**Affiliations:** ^1^ Shaanxi Key Laboratory for Animal Conservation, College of Life Sciences Northwest University Xi'an Shaanxi China; ^2^ School of Medicine Northwest University Xi'an Shaanxi China

**Keywords:** extra‐pair paternity, mammalian mating systems, reproductive strategy, reproductive success, social evolution

## Abstract

In social mammals, dominance status and tenure length are often important determinants of male reproductive success. Nevertheless, alternative strategies, such as extra‐pair mating, and the active role of females in securing reproductive benefits can substantially modify patterns of paternity. To date, many studies have had short observation periods and small sample sizes, constraining understanding of how male social status, tenure length, and female mating strategies jointly affect male long‐term reproductive success, especially in species with complex social systems. Using longitudinal demographic and genetic data from a free‐ranging population of a golden snub‐nosed monkey ( *Rhinopithecus roxellana* ) multilevel society in the Qinling Mountains, central China, we investigated how leader males' ranks and tenure lengths influence their reproductive success through within‐unit and extra‐unit paternities. We found that although high rank increases the likelihood of producing within‐unit paternity offspring in the short term, tenure length is a stronger determinant of long‐term reproductive success via both within‐unit and extra‐unit paternities. Females may gain direct benefits from producing extra‐pair paternity offspring by reducing the risk of infanticide through mating with all‐male band males and/or by selecting high‐ranking leader males for better access to resources. Moreover, females may also accrue additional indirect benefits from producing extra‐pair paternity offspring when more adult males are available in the breeding band. Our findings highlight extra‐pair paternity as a reproductive strategy for both male and female golden snub‐nosed monkeys to optimize reproductive success, which may also play a role in maintaining stability in this complex primate society.

## Graphical abstract

This paper is the latest study to explore the effects of social status and reproductive strategy intake on the males' long‐term reproductive success in a multilevel society using longitudinal demographic and genetic data. The findings of this study further expand the significance of extra‐pair paternity as a reproductive strategy, revealing how social species balance intersexual conflict in comparative studies of reproductive success.

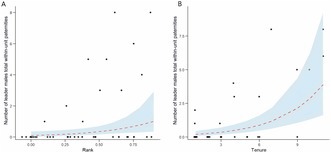

## 1 Introduction

### 1.1 Sexual Selection and Mating Strategies

Animal mating systems are shaped by a combination of ecological factors and sex differences in mating frequency (Andersson 1994; Emlen and Oring 1977). Ecological conditions influence the distribution and availability of mates, while sex differences in reproductive strategies largely stem from differences in parental investment (Trivers 1972). These asymmetries in reproductive investment give rise to inevitable sexual conflict, as males and females pursue divergent strategies to maximize their own reproductive success (Chapman et al. 2003; Parker 1979). This conflict results in the evolution of distinct reproductive strategies for each sex as both males and females attempt to maximize their own reproductive success (Aloise King et al. 2013; Arnqvist and Rowe 2005; Stumpf et al. 2011).

In most mating systems, both males and females exhibit varying degrees of polygyny/polyandry, respectively (Birkhead and Pizzari 2002). It is often clear why males mate with multiple females and also typically aim to monopolize access to reproductively active females; reproductive success generally increases with the number of females inseminated (Bateman 1948). Although the benefits of male polygyny are often direct and conspicuous, the advantages of female polyandry are equally important but can be more nuanced, generally categorized into either direct benefits to females or indirect genetic benefits to their offspring (Jennions and Petrie 2000; Wolff and Macdonald 2004).

### 1.2 Social Structure and Constraints on Reproductive Success

How these potential benefits become realized to members of either sex depends largely on the social structure and the mating system. For example, in species in which adult males and females associate at a single location over a limited reproductive time period, male–male competition can be intense, with very few dominant males fathering most offspring (Grebe et al. 2022; Le Boeuf 1974; Miller et al. 2016; Lemaître et al. 2014; Wroblewski et al. 2009). Most males thus achieve little or no reproductive success, with females having limited opportunities to choose the father of their offspring. Conversely, when males cannot defend large numbers of females, male reproductive success may be less skewed, with females able to more freely control the paternity of offspring (Birkhead and Møller 1993).

In species with highly complex social systems such as those with a multilevel society (MLS) such as hamadryas baboons, geladas, and golden snub‐nosed monkeys, females may engage in mating with more than one male at different hierarchical levels, potentially gaining genetic and/or social benefits (Snyder‐Mackler, Beehner, and Bergman 2012; Zhang et al. 2023). Most MLSs are characterized by the one‐male unit (OMU), in which one adult ‘leader’ male socializes mainly with several adult females, with several OMUs collectively forming a larger group, the breeding band (Grueter et al. 2020). Females can produce offspring not only with their OMU leader male but also as a result of mating with either other OMU leader males, or with those adult males excluded from the breeding band that may opportunistically shadow the breeding band for access to females (males of the all‐male band). Females can thus benefit indirectly by producing offspring that have variable genotypes with potentially enhanced adaptability to environmental perturbations (Bichet et al. 2016; Reed and Frankham 2003; Szczecińska et al. 2016) or increased immunocompetence (Hamilton and Zuk 1982; Johnsen et al. 2000; Zhang et al. 2023).

Additionally, females may obtain direct benefits from mating with and producing offspring fathered by multiple males (Nichols et al. 2015). For example, females may gain due to obtaining extra food (Tryjanowski and Hromada 2005), protection against predators (Gray 1997), increased paternal care (Goldizen 1987; Townsend et al. 2010) and a reduction in infanticide risk due to paternity uncertainty (Hrdy 1979; Lukas and Huchard 2014). It is widely accepted that females may prefer males who offer indirect genetic benefits, thereby enhancing the genetic quality of their offspring (Brouwer and Griffith 2019). In contrast, the role of direct benefits remains less well understood, largely due to the lack of clear and quantifiable evidence in many species.

In species that live in multilevel societies, competition can also result in the OMU leader males forming a dominance hierarchy within the breeding band, in which high‐rank males and by default other members of their OMU gain privileged access to prime resources such as food (Guo et al. 2020; He et al. 2025). Social rank and length of tenure, the time a leader male maintains his social status, are important determinants of male reproductive success (Duncan et al. 2023) and survival (Cram et al. 2018; Mitani et al. 2012; Snyder‐Mackler et al. 2020; Taborsky 2021). Once a male attains leader male status, usually by usurping another leader male, he gains access to females in ‘his’ new OMU and the wider breeding band (Clutton‐Brock 1991; Packer et al. 1990). Protracted tenure may also allow males to strengthen social bonds with the females of their OMU (Orbach 2019), who may also benefit by having privileged access to resources (He et al. 2025). Achieving high social rank may thus further increase male mating opportunities, if females in their own OMU are less likely to engage in matings with other males and/or if females from other OMUs are more likely to engage in matings with high‐rank leader males, which may also vary according to the time dominant individuals maintain their status (Clutton‐Brock 1988; Clutton‐Brock et al. 2006; Lardy et al. 2015).

### 1.3 Evidence From Other Social Mammals

Studies of various species of social mammals have found a complex interplay between male dominance, male tenure length, and female choice in determining male reproductive success. For example, in bats (Mccracken and Wilkinson 2000; Popa‐Lisseanu and Voigt 2009), geladas (Beehner and Bergman 2008; Grueter et al. 2017), red deer (*Cervus elaphus L*.) (Clutton‐Brock 1985, 1988; Clutton‐Brock et al. 1982), and northern elephant seals ( *Mirounga angustirostris* ) (Le Boeuf et al. 2019), dominant males with longer tenures typically achieve high reproductive outputs. However, female reproductive strategies such as mating with dominant and subordinate males can counterbalance the effects of dominant males who attempt to monopolize reproduction within the social group. In East African blue monkeys ( *Cercopithecus mitis* ), female social behaviors play an important role in shaping male mating opportunities and long‐term reproductive success (Cords 2012; Roberts et al. 2014). These insights emphasize the importance of considering both male social parameters and female mating strategies in shaping reproductive patterns in socially complex species.

### 1.4 The Golden Snub‐Nosed Monkey

**FIGURE 1:**
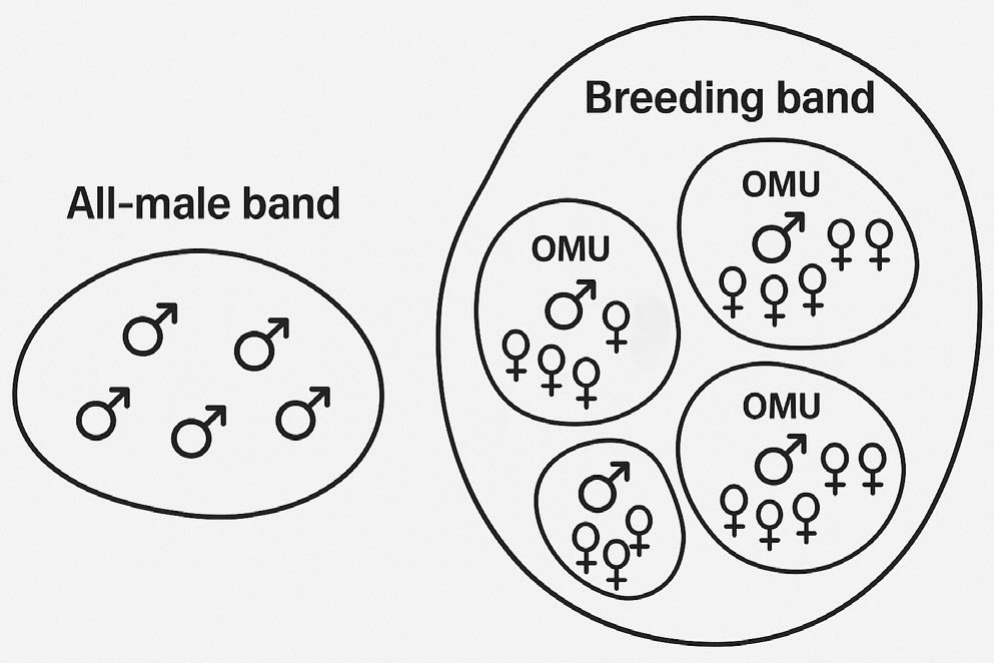
The multilevel social organization of the Golden snub‐nosed monkey.

The golden snub‐nosed monkey ( *Rhinopithecus roxellana* ) is an ideal model species for investigating how sexual strategies of both sexes and male social status affect reproductive skew within a social group. This species has a multilevel society (see Figure 1), in which several OMUs, each composed of a single adult leader male, 1–7 adult females, and their dependent offspring, collectively form a breeding band (Qi et al. 2014). In contrast to female transfers, which often occur between OMUs within the breeding band, OMUs themselves are typically formed when newly immigrated males from bachelor groups displace former leader males through takeovers to establish new social units (Huang et al. 2017; Inoue and Takenaka 2008; Qi et al. 2014). The adults within each OMU tend to socialize and coordinate their daily activities together rather than with other OMUs (Grueter et al. 2017; Qi et al. 2017). The breeding band is shadowed by an all‐male band, comprising the sub‐adult and adult males that have been excluded from the breeding band and periodically attempt to usurp OMU leader males (Fang et al. 2019; Qi et al. 2017).

Although females solicit mating during both fertile and non‐fertile periods, golden snub‐nosed monkeys are strictly seasonal breeders (Qi et al. 2008). The mating season typically occurs from late September to December, with all births taking place during the following March to May (Qi et al. 2009). Gestation lasts approximately 6–7 months. Females generally give birth every 2 years; however, if an infant dies within 6 months of birth, the mother may resume ovulation in time to conceive during the next breeding season (Qi et al. 2008). There is evidence of extra‐pair matings and paternities in wild *R. roxellana* between adult females and males of different OMUs from those of the females and with all‐male band males (Guo et al. 2010; Qi et al. 2020). However, patterns of reproductive skew among OMU males and between OMU and all‐male band males have been shown to vary (Guo et al. 2010; Zhang et al. 2020), with no previous study also including the effects of leader male social rank and tenure using long‐term datasets.

Moreover, although there are known indirect genetic benefits to female *R. roxellana* through producing offspring of mixed paternities (Zhang et al. 2023), the importance of direct material benefits remains relatively unexplored (but see Qi et al. (2020)). Any direct benefits to females producing mixed‐paternity offspring may relate to OMU rank and tenure length, which thus need to be included when measuring male reproductive skew in the breeding band.

### 1.5 Study Aims and Predictions

Over 15 consecutive years we studied a wild *R. roxellana* breeding band, accumulating data for social structure, the dynamics of the OMU leader male social hierarchy, and the paternities of all offspring born during the study period. These data provided the basis for quantifying both the reproductive skew among leader males in the breeding band and the proportion of extra‐pair paternity offspring in each OMU, sired by either other OMU leader males or males from the all‐male band.

To understand the determinants of leader males' reproductive success and extra‐pair paternity in OMUs, we assessed the effects of leader male rank and tenure length. We formulated four key predictions to guide our analysis. (1) Both high rank and lengthy tenure result in increased overall leader male reproductive success through the production of offspring with the females of his own and from other OMUs, and hence reproductive skew will be biased towards high‐rank males. This pattern arises because females from other OMUs may gain direct benefits through increased access to resources by associating and producing extra‐pair offspring with a high‐ranking leader male from a different OMU to her own, and the females of high‐ranking OMUs may have reduced propensity to produce extra‐pair paternity offspring. (2) Leader male rank is negatively correlated with the number of extra‐pair offspring within his OMU. (3) If females produce offspring fathered by males other than the leader male of their own OMU, due to direct benefits from infanticide avoidance by paternity confusion, such offspring should predominantly be fathered by all‐male band males, not the leader males of other OMUs. Moreover, paternity confusion should also result in: (4) a positive correlation between the number of offspring fathered by other leader males within each OMU and the number of OMUs in the breeding band (more leader males). This pattern would result from increased female choice due to a larger sampling pool of OMU leader males and an increased likelihood of OMU usurpation by all‐male band males.

## 2 Materials and Methods

### 2.1 Field Research

The golden snub‐nosed monkeys of the Zhou‐National Nature Reserve (ZNNR) (108°14′–108°18′ E, 33°45′–33°50′ N) in the northern Qinling Mountains, Shaanxi Province, central China, have been studied continuously over the past 20 years. To enable close observation and individual identification, the study breeding band has been semi‐provisioned since October 2001. Each monkey in the population can be identified by pelage color, body size and other unique physical characters. We determined the social structure of this breeding band by recording the group size, the composition of each OMU, and the social rank of each OMU leader male every year from 2001 to 2015. We also took a total of 489 genetic samples (191 hair samples and 298 fecal samples, including individual replicates over the years which allowed us to replicate genotyping experiments and correct potential errors) involved only non‐invasive sample collection to enable us to determine the paternities of all offspring produced during the study period (see Zhang et al. (2023) for details).

### 2.2 Paternity Analyses

In order to obtain accurate paternity data, each hair and fecal sample was sequenced (see below) and any duplications excluded. All offspring samples were subject to paternity testing. When the father was the leader male of the offspring mother's OMU, the individual was classified as the result of within‐unit paternity. In cases in which the father was not the leader male of the offspring mother's OMU, the individual offspring was categorized as the result of extra‐pair paternity (i.e., the father was either another OMU leader male or an all‐male band male). The total reproductive output for leader males was thus the sum of the offspring fathered with females within their own OMUs (within‐unit paternities) and the offspring fathered with females who were members of other OMUs (extra‐unit paternities). Therefore, at any one time, the dependent offspring within each OMU potentially had all within‐unit paternities, all extra‐unit paternities, or a combination of both within‐ and extra‐unit paternities. Extra‐unit paternity offspring could have been fathered by either another OMU leader male or an all‐male band male.

Each DNA sample was amplified with 19 polymorphic microsatellite loci (Table S1) on an ABI Veriti thermocycler using the following program: incubation at 95°C for 5 min, followed by 30 cycles (94°C for 30 s, 55°C–60°C for 45 s, 72°C for 45 s) and 72°C for 10 min. Alleles were separated by capillary electrophoresis using an ABI PRISM 3100 Genetic Analyzer, and the size of the alleles relative to an internal size standard (ROX‐labeled HD400) was determined using GENEMAPPER v. 3.7 (Applied Biosystems, Foster City, CA, USA). To prevent genotyping errors such as false alleles and allelic deletions, pure heterozygote genotypes were confirmed by five independent replicates, and all observed heterozygotes were confirmed by at least seven separate reactions (Teichroeb and Jack 2017).

Based on the 19 microsatellite loci data, we used the Identity Analysis function in CERVUS v3.0 (Kalinowski et al. 2007) for individual sample identification. The program Parentage Analysis was used to identify the paternity of each offspring under the following conditions: 10,000 simulation cycles, 100% locus input, 1% error rate, and 80% and 95% confidence levels. Paternity was determined by further checking loci in paternity‐infant‐maternity triplet genetic relationships (Modolo and Martin 2008). For paternity determination, we allowed for one mismatch at all loci in each parent‐offspring pair to account for possible mutations (Pemberton et al. 1992). A Hardy–Weinberg equilibrium test (Rousset 2008) was performed for each microsatellite locus using GENEPOP V4.3, with a Bonferroni correction (Rice 1989). No deviation from Hardy–Weinberg expectations was found for any of the genotypes in the population. Therefore, we isolated 139 alleles from 19 microsatellite loci, with an allele range of 4–9 at each locus (mean allele value 7.32 ± 1.13), an apparent heterozygosity of 0.67 ± 0.10, and an expected heterozygosity of 0.60 ± 0.10 in order to identify the paternity of each offspring.

### 2.3 OMU Leader Male Rank and Tenure

During all observation periods, we systematically recorded both agonistic and submissive behaviors to assess dominance relationships among OMU leader males (Fang et al. 2022; He et al. 2025). We determined agonistic interactions as those in which one male (the initiator) threatened another (the receiver) by leaning forward, showing its teeth and staring directly or chasing the receiver. During or after such a chase the initiator almost always made contact and grappled with the receiver. Data for antagonistic interactions that escalated and ultimately involved more monkeys than two OMU leader males were not included. For each year of the study, to determine the social rank we calculated a David's score for each OMU leader male (NDS) (David 1987). This measure is based on the proportions of ‘wins’ combined with an unweighted and a weighted sum of its proportions of ‘losses’ in dyadic contests, usually due to a dispute over an individual food item, with another OMU leader male (Fang et al. 2022). Given year‐to‐year variation in the number of OMUs and hence leader males in the breeding band, rank number of the same males varied between years. Leader males were assigned ‘yearly ranks’ according to the proportion of leader males dominated during each year (relative rank) (Silk et al. 2010). The proportion of leader males dominated was calculated as (*N* − *d*)/(*N* − 1), in which *N* is the total number of leader males in the breeding band each year and *d* is the ordinal rank of a particular leader male. The highest‐ranking leader male is ranked 1, and the lowest‐ranking leader male is ranked 0.

We also calculated an alternative measure for rank, which used the mean values of yearly rank as an ‘average rank’ over the total study duration. In relatively long‐lived iteroparous species, individual longtime reproductive success results from an accumulation of reproductive events over an entire lifespan. The social status of leader male *R. roxellana* in the breeding band is relative to other leader males in the breeding band at any one time, and may change over time. Thus, the use of average rank is able to more accurately reflect the effects of leader male social status over the course of the study when predicting reproductive success.

Here, tenure length refers to the number of years over which a male has remained as a leader male of his OMU in this breeding band. At the beginning of the study, all OMU leader males were assigned a tenure of 1 year because demographic and genetic data were all collected for the initial year of the study. Hiatuses in tenure due to males leaving and then rejoining the breeding band during the study were incorporated into their tenure lengths.

### 2.4 Reproductive Skew and Paternity Distributions

To partially test prediction one, we assessed bias in the distribution of all offspring paternities among OMU leader males within the study breeding band by calculating Nonacs's binomial skew index *B* (Nonacs 2003) using the software SKEW. Nonacs's index *B* ranges from −1 to +2; positive values indicate that skew is greater than expected, with negative values indicating that skew is less than expected, such that reproduction is relatively evenly distributed. *B* = 0 indicates that paternities are randomly distributed among potential fathers (Holekamp et al. 2012). To test prediction three, we examined the numbers and proportions of offspring that were the result of within‐ or extra‐unit paternities attributed to either OMU leader males or all‐male band males at the time of their conception.

### 2.5 Statistical Analyses

To evaluate the associations between leader male rank, tenure length, and the number of adult females in each OMU, we used the “ppcor” package in R 4.0.4 (Team RCTC 2021) to calculate partial Pearson correlation coefficients (“pcor.test” function) (Kim 2015). This enabled us to calculate each pairwise correlation coefficient independent of a third variable. We found significant partial correlations between rank and tenure length (*r*_p_ = 0.43, *p* < 0.001) and between rank and the number of adult females in an OMU (*r*_p_ = 0.42, *p* < 0.001). The correlation between tenure length and the number of adult females in an OMU was not significant (*r*_p_ = −0.14, *p* = 0.17). Given the significant associations between the number of adult females and leader male rank and tenure length, we omitted the number of adult females in each OMU as an explanatory variable from statistical models to simplify model structure and exclude inflation of estimates due to multicollinearity.

To further test prediction one, and to test predictions two and four, we used a series of generalized linear models (GLMs) and generalized linear mixed models (GLMMs) (Baayen 2008). We used five models to describe: (1) the number of within‐unit offspring fathered by each OMU leader male in each year of the study (yearly within‐unit paternity, individual level), (2) the number of extra‐unit paternity offspring fathered by each OMU leader male in each year of the study (yearly extra‐unit paternity, individual level), (3) the total number of within‐unit paternity offspring fathered by each OMU leader male over the entire study period (total within‐unit paternity, individual level), (4) the total number of extra‐unit paternity offspring fathered by each OMU leader male over the entire study period (total extra‐unit paternity, individual level), and (5) the number of extra‐pair paternity offspring within each OMU for each year of the study (yearly extra‐pair paternity, OMU level). Models 1, 2, and 5 used offspring paternity data that were further categorized by year, whereas models 3 and 4 used total reproductive success over the entire 15‐year study period.

For models 1 and 2, as fixed explanatory variables we used OMU leader male ‘yearly rank’, tenure length and their interaction. In models 3 and 4, fixed explanatory variables were OMU leader male ‘average rank’ over the entire study period, tenure length and their interaction. For model 5, as fixed explanatory variables we used OMU leader male ‘yearly rank’, tenure length and their interaction, and the number of OMUs in the breeding band in each year of the study. Models 1, 2 and 5 were GLMMs and included both ‘leader male ID’ and ‘year’ as random effects, whereas models 3 and 4 were GLMs. We also fitted five additional models that were each identical in construction except that the rank × tenure length interaction was omitted (see Supporting Information). Initial analysis using models each assuming a Poisson distribution suggested data were under‐dispersed. For each model we therefore used a Conway‐Maxwell‐Poisson (CMP) distribution and a log link function, which is suitable for both GLMs and GLMMs when data are either under‐ or over‐dispersed (Brooks et al. 2019). Each GLMM was fitted using the “glmmTMB” function of the “glmmTMB” package (Brooks et al. 2017) for R 4.0.4 (R Core Team 2013). After analysis, we tested for under‐ and over‐dispersion in the residuals of all models using the “testDispersion()” function in the R package “DHARMa” (Hartig 2018). We also tested if the expected number of zeros in our response variable based on a fitted model differed to the observed number of zeros, using the “testZeroInflation()” function of “DHARMa” (Hartig 2018). We found no evidence for data under‐ or over‐dispersion nor for zero expansion. Collinearity was also not an issue as indicated by the variance inflation factors (VIF) of each model (Quinn and Keough 2002). By using the function “check_collinearity()” of the R package “performance” (Lüdecke et al. 2019) we found no evidence for multicollinearity.

For our GLMMs, we first determined the statistical significance of the full model by comparing its fit to that of the null model (comprising only the random effects) by applying likelihood ratio tests (R function “anova”) (Dobson and Barnett 2018). For all models, we assessed the significance of each explanatory term, including the rank × tenure length interaction, using likelihood ratio tests (R function “drop1”). We considered *p* values < 0.05 as significant and 0.05 < *p* < 0.1 as a trend (Stoehr 1999).

## 3 Results

From 2001 to 2015, we recorded a total of 40 OMU leader males and 142 adult females in the breeding band. The number of OMUs per year ranged from 7 to 14, and the number of females per OMU ranged from 0 to 9 (mean ± SD = 3.98 ± 1.62) (Table S2). Although OMUs generally include one male and multiple females, we retained the few transitional ‘zero‐female’ OMUs that temporally existed during the study in the dataset (e.g., after female dispersal or before new unit formation), because the resident males remained spatially and socially integrated within the breeding band. The tenure lengths of the OMU leader males over the study period ranged from one to 11 years (mean ± SD = 3.38 ± 3.09 years), with 11 males having tenures 5 years or longer.

### 3.1 Reproductive Skew and Paternity Distributions

Over the 15‐year study period, we found significant reproductive skew among OMU leader males (*B* = 0.023, *p* < 0.01). Three‐quarters (75%, *N* = 30) of all males (*N* = 40) that achieved OMU leader male status fathered at least one offspring, with 10 (25%) failing to father any offspring. The mean offspring fathered per male (the sum of within‐unit and extra‐unit offspring) was 3.03 ± 4.64. Four males fathered a total of 63 offspring (range of 12–21), 49.6% of all offspring (*N* = 127) born into the breeding band (Table S3). Of these 63 offspring, 27 (42.9%) were with‐unit offspring fathered by the leader males of their mothers' OMUs. The remaining 36 (57.1%) were extra‐pair offspring fathered by males that were not the leader males of their mothers' OMUs; their fathers were thus other OMU leader males or all‐male band males (Table S3).

Of the 120 paternities we successfully determined, 46 (38.3%) were within‐unit offspring fathered by the leader males of the same OMUs as that of the mothers. The remaining 74 (61.7%) paternities were extra‐pair offspring fathered by leader males from different OMUs to that of the mothers or all‐male band males (Table 1 and Table S3). A more detailed examination of the 74 extra‐pair paternity offspring that could not be attributed to the leader males of the mothers' OMUs showed that 43 (58.1%) were fathered by OMU leader males that maintained their leader status throughout the duration of the study (Table 1). However, the remaining 31 extra‐pair paternity offspring were fathered by males who, at the time of conception, were members of the all‐male band but later entered the breeding band and achieved leader male status during the study period. Furthermore, of these 31 offspring, 20 (64.5%) were fathered by all‐male band males who subsequently entered the breeding band; nine (29%) were fathered by males who had formally held OMU leader status in the breeding band and had been ejected, with two (6.5%) fathered by males who had been OMU leader males in the breeding band both before and after the year of conception (Table 1). Overall, at the time of conception, 89 (70.1%) offspring were fathered by an OMU leader male, with 38 (29.9%) being fathered by an all‐male band male (Table 1 and Table S3).

**TABLE 1:** Summary of paternity assignments.

Category	Count	Percentage
Total assigned paternities	120	100
Within‐unit paternities	46	38.3
Extra‐pair paternities	74	61.7
By OMU leader males (during entire study)	43	58.1
By all‐male band males (at conception)	31	41.9
Later became OMU leaders	20	64.5
Previously held OMU leadership (ejected)	9	29
Held leadership before & after conception	2	6.5
Summary by status at conception		
Offspring fathered by OMU leader males	89	70.1
Offspring fathered by all‐male band males	38	29.9

### 3.2 Effects of Rank and Tenure Length on Paternities

**FIGURE 2:**
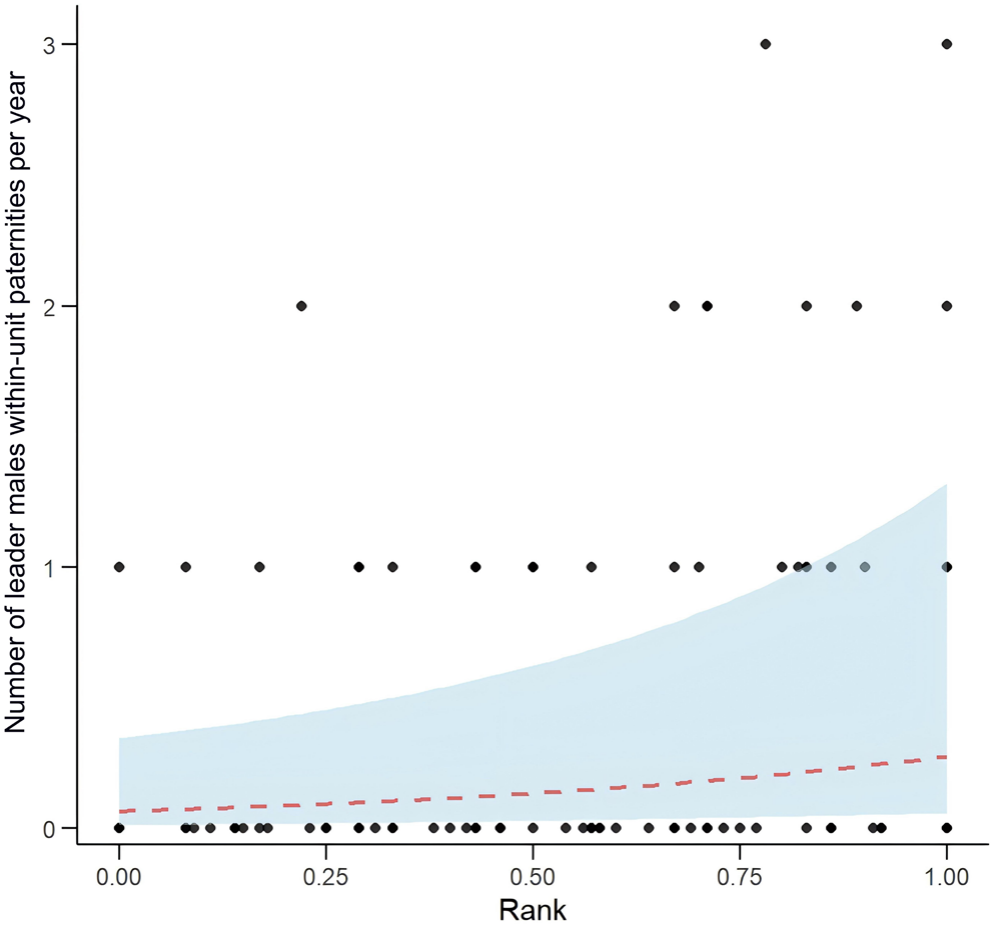
Relationship between yearly rank and within‐unit paternity of leader males. *Points* show the raw data, the *lines* indicate the fitted model (*dashed*, *red*) with the surrounding 95% credible interval (*area*, *light blue*). The plot demonstrates that higher‐ranked males tend to father more within‐unit offspring per year.

Model 1 showed that the number of within‐unit paternity offspring fathered by individual leader males during each year increased significantly with ‘yearly rank’ (*χ* ^2^ = 7.200, *p* = 0.007), an effect not dependent on the length of tenure, with tenure not significantly affecting the number of within‐unit offspring produced (*χ* ^2^ = 0.079, *p* = 0.778) (Table 2 and Table S4; Figure 2).

**TABLE 2:** Summary of five models evaluating predictors of male reproductive success (full model, unless denoted otherwise).

Models	Predictor variable	GL(M)Ms			Likelihood ratio tests		
		95% CI	Estimate	SE	*χ* ^2^	df	*p*
Model 1: Yearly within‐unit paternity (individual male level)	Intercept	[−2.802, −1.092]	−1.947	0.436			
	Tenure	[−0.159, 0.119]	−0.020	0.071	0.079	1	0.778
	Yearly rank	[0.441, 2.463]	1.452	0.516	7.200	1	0.007
Model 2: Yearly extra‐unit paternity (individual male level)	Intercept	[−2.620, −1.072]	−1.846	0.395			
	Tenure	[−0.012, 0.192]	0.090	0.052	2.948	1	0.086
	Yearly rank	[−0.697, 1.231]	0.267	0.492	0.294	1	0.588
Model 3: Total within‐unit paternity (individual male level)	Intercept	[−4.411, −1.545]	−2.978	0.731			
	Tenure	[0.176, 0.420]	0.298	0.062	18.390	1	< 0.001
	Average rank	[0.459, 4.857]	2.658	1.122	5.475	1	< 0.001
Model 4 (additional model): Total extra‐unit paternity (individual male level)	Intercept	[−1.778, 0.614]	−0.582	0.610			
	Tenure	[−0.631, 0.181]	−0.225	0.207			
	Average rank	[−4.086, 0.924]	−1.581	1.278			
	Tenure × Average rank	[0.202, 1.428]	0.815	0.313	5.499	1	0.019
Model 5: Yearly extra‐pair paternity (OMU level)	Intercept	[−1.681, 0.361]	−0.660	0.521			
	Tenure	[−0.193, 0.012]	−0.090	0.052	3.091	1	0.079
	Yearly rank	[−0.542, 0.841]	0.149	0.353	0.178	1	0.672
	N_OMUs_	[0.006, 0.173]	0.089	0.043	3.893	1	0.048

*Note:* Within‐unit paternity refers to offspring fathered with females within the leader males' own OMUs. Extra‐unit paternity refers to offspring fathered with females outside the leader males' own OMUs. Extra‐pair paternity refers to offspring fathered by other OMU leader males or all‐male band males. Models 1–4 are at the individual leader male level; Model 5 is at the OMU level.

Abbreviation: OMUs, one‐male units.

For Model 2, both tenure (*χ* ^2^ = 2.948, *p* = 0.086) and ‘yearly rank’ (*χ* ^2^ = 0.294, *p* = 0.588) did not significantly affect the number of extra‐unit paternity offspring (Table 2 and Table S5).

**FIGURE 3:**
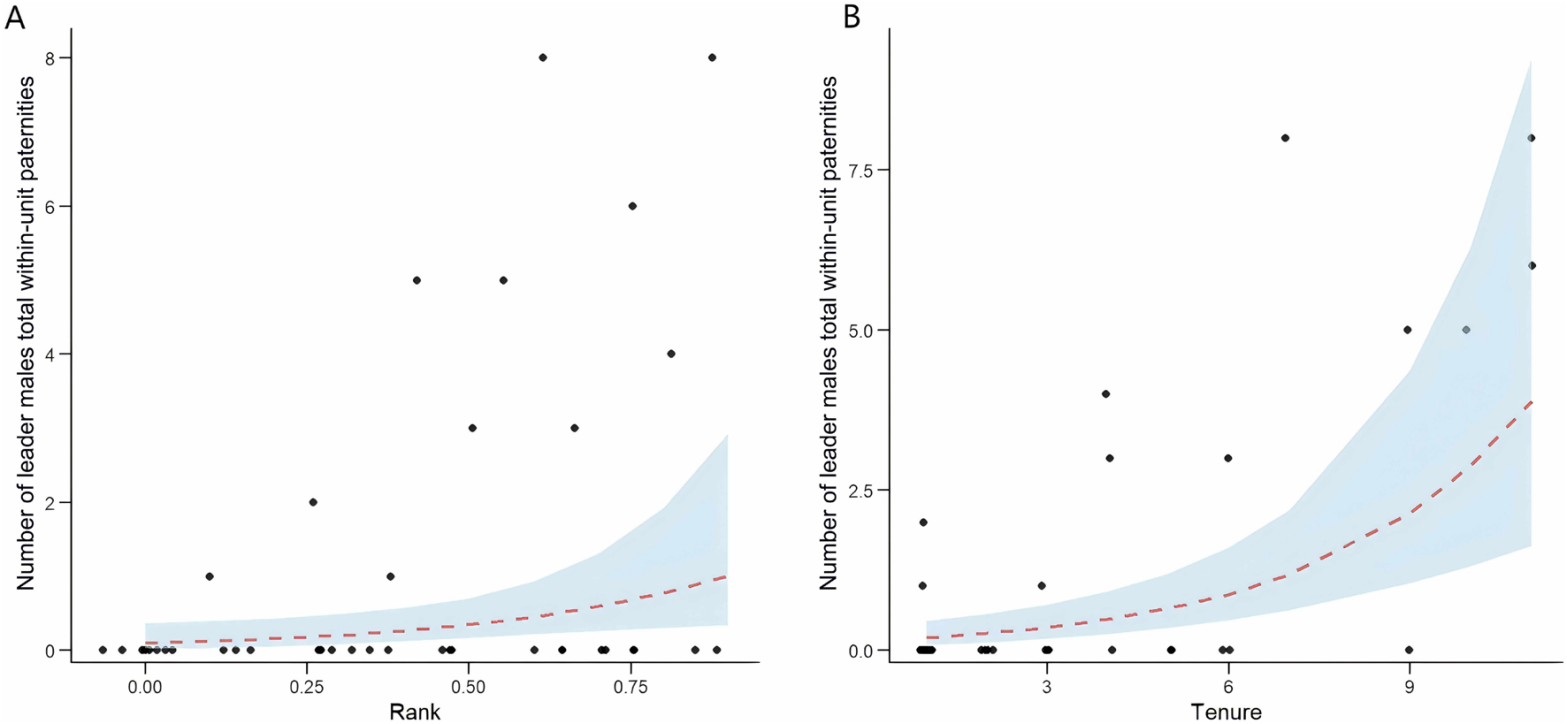
Effects of average rank (A) and tenure length (B) on total within‐unit paternities over the 15‐year study period. *Points* show the raw data, the *lines* indicate the fitted model (*dashed*, *red*) with the surrounding 95% credible interval (*area*, *light blue*). Panel A shows that males with higher average rank tend to achieve greater total within‐unit reproductive success. Panel B indicates a positive association between tenure length and within‐unit paternity.

When OMU leader male rank and reproductive output were assessed across the complete 15‐year study period, Model 3 showed that the number of within‐unit paternity offspring produced by each OMU leader male increased significantly with both ‘average rank’ (*χ* ^2^ = 5.475, *p* < 0.001) and tenure length (*χ* ^2^ = 18.390, *p* < 0.001). However, the ‘average rank’ × tenure length interaction was not significant (Table 2 and Table S6). The result shows that achieving a high overall rank or maintaining OMU leader status for a protracted time period in the breeding band translated directly to the production of within‐unit paternity offspring (Figure 3).

**FIGURE 4:**
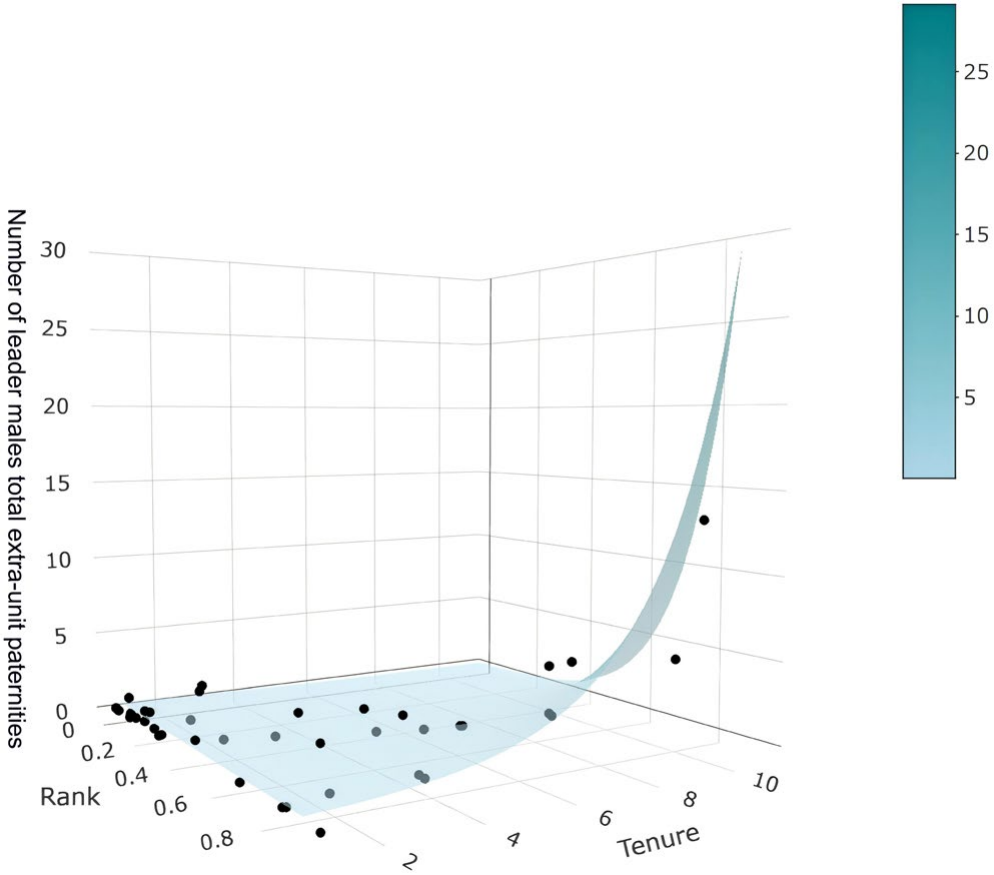
3D interaction between tenure length and rank on leader males' total extra‐unit paternities. This surface plot illustrates a model‐fitted trend showing that tenure length increases extra‐unit reproductive success predominantly among higher‐ranking males.

Model 4 similarly examined the number of extra‐unit paternity offspring produced by OMU leader males over the entire study period. ‘Average rank’ had no significant independent effect, but tenure length showed a marginal trend. The interaction between ‘average rank’ and tenure was significant (*χ* ^2^ = 5.499, *p* = 0.019), showing that as OMU leader rank increased with tenure, the number of extra‐pair offspring produced increased (Figure 4; Table 2 and Table S7).

Model 5, which used year‐by‐year data, showed that both ‘yearly rank’ (*χ* ^2^ = 0.178, *p* = 0.672) and tenure (*χ* ^2^ = 3.091, *p* = 0.079) failed to significantly predict the number of extra‐pair paternity offspring within each OMU. However, the negative trend for tenure length suggested that males with shorter tenures were more likely to preside over OMUs containing more offspring of extra‐pair paternity, that is, those fathered by other males. Importantly, OMU number had a significant positive effect on the number of extra‐pair paternity offspring (*χ* ^2^ = 3.893, *p* = 0.048) (Table 2 and Table S8); the more OMUs present in the breeding band as a whole, the more extra‐pair paternity offspring were present within each OMU.

## 4 Discussion

The *R. roxellana* social structure and mating system results in significant reproductive skew among OMU leader males, and high rates of extra‐pair and extra‐unit offspring paternities. Of the 40 adult males who achieved OMU leader status, four fathered 49.6% of all offspring born during the 15‐year study period. Our results also show that extra‐unit paternities form a substantial part of OMU leader male reproductive success. High rates of extra‐pair paternities showed that offspring within OMUs are often fathered by other OMU leader males or males of the all‐male band, with approximately one‐third of all offspring at the time of conception being fathered by an all‐male band male, most of which subsequently entered the breeding band and achieved OMU leader male status. Our results are largely consistent with adult males that achieve OMU leader status and then remain in their positions for protracted time‐lengths furthering their reproductive success via increased access to females. At the same time, females further their reproductive interests by producing offspring with their OMU leader male, with other OMU leader males and all‐male band males likely obtaining both direct and indirect benefits as a result.

### 4.1 Social Rank

Previous studies have found positive relationships between social dominance and reproductive success in both sexes across multiple mammal and bird species (Ilany et al. 2021; Stoinski et al. 2009; Surbeck et al. 2017; Tibbetts et al. 2022; Wood et al. 2022; Wroblewski et al. 2009). We found some evidence that rank translated into increased OMU leader male reproductive success through fathering offspring with females within their own OMUs but not with the females of other OMUs. OMU leader males of high rank have increased direct access to adult females because their OMUs contain more females than those of lower‐ranking males. However, it appears that these potential benefits to high‐rank males may be partially offset by females within their OMUs producing offspring fathered by other males. In *R. roxellana* , high rank therefore does not entirely explain male reproductive success via increased access to females, as has been found in other mammal species (Cowlishaw and Dunbar 1991; Ellis 1995; Rodriguez‐Llanes et al. 2009).

High‐rank OMU leader males (Guo et al. 2020) and the adult females of their OMUs (He et al. 2025) obtain privileged access to food resources, especially during winter. By securing privileged access to food resources, adults of high‐ranking OMUs are able to spend less time feeding and devote more time to behaviors associated with promoting social cohesion within the unit, such as grooming (Guo et al. 2020). We predicted that high‐rank OMU leader males would produce more extra‐unit paternity offspring than lower‐rank males. By mating with high‐rank males, females from other OMUs may directly benefit from increased access to food resources or by producing genetically variable offspring of uncertain paternity and/or of increased genetic fitness. However, we found no evidence of male rank affecting the production of extra‐unit paternity offspring. This lack of association indicates that, although both males and females benefit from high social rank, benefits to males are mainly accrued through access to the higher numbers of females in their OMUs rather than a reduced propensity for the females within their own OMUs to produce extra‐pair offspring with other males or by fathering extra‐unit offspring with the females from other OMUs.

### 4.2 Length of Tenure

Consistent with our prediction, the longer an adult male maintained OMU leader status within the breeding band, the higher his reproductive success, through the production of both within‐ and extra‐unit paternity offspring. In vertebrate species with high reproductive skew, the length of time that individuals hold positions of dominance often correlates with their long‐term reproductive success (Clutton‐Brock 1988; Robbins et al. 2011; Rossiter et al. 2006). However, maintaining high rank also comes with costs such as increased physiological stress resulting in reduced foraging, immune capability, and metabolic function (Hoffman et al. 2010; Muehlenbein and Watts 2010; Muller and Wrangham 2004; Sapolsky 2005), increased rates of energy consumption (Georgiev et al. 2015), and increased likelihood of involvement in intra‐sexual antagonistic contests (Tibbetts et al. 2022).

Our results suggest that in this *R. roxellana* breeding band, as long as a male maintains his OMU leader status and remains within the breeding band, this enables him to reproduce with (some of) the females of his own and other OMUs. Although high rank gives OMU leader males clear material (Guo et al. 2020) and reproductive benefits (via within‐unit paternity offspring), explaining the rank dynamics due to competition among leader males and between leader males and all‐male band males during the course of the study, leader male status does not always result in reproductive success (Ellis 1995; Huchard et al. 2016; Roberts and Cords 2015; Roberts et al. 2014; Surbeck et al. 2017); a quarter of all leader males (10 individuals) failed to produce any offspring during the study. Furthermore, more detailed examination of the long‐term status dynamics of individual males, for instance the time spent in the all‐male band, the nature of how they achieved OMU leader male status, and their rank changes after entering the breeding band is therefore required.

Our results show an interplay between tenure length and rank on overall number of extra‐unit paternities. The interaction between tenure length and rank infers a non‐additive relationship in which the influence of tenure length on reproductive success is contingent on rank. Males with higher rank derive increased reproductive benefits from longer tenure, benefits generally unavailable to lower‐rank males. Thus, the tenure length × rank interaction captures a nuanced effect that shows that male reproductive success is maximized when both tenure length and rank are high, reflecting a hierarchical and temporal advantage in male reproductive strategies.

### 4.3 Cooperation Among Adult Male *R. roxellana* May Help Optimize Their Reproductive Success

In *R. roxellana* , OMU leader males in the breeding band collectively defend their reproductive interests from all‐male band males (Xiang et al. 2014), with all‐male band males forming kinship alliances to periodically attempt to usurp leader males (Qi et al. 2017). The paternity distributions in our study breeding band suggest that these leader male tactics, although likely to reduce usurpations and hence help prolong individual tenure length, do not also function to effectively minimize the number of extra‐pair paternity offspring within OMUs.

The OMU leader males within an *R. roxellana* breeding band are predominantly in a state of mutual tolerance, a prerequisite for the long‐term stability of their multilevel society (Grueter et al. 2012, 2017). This mutual tolerance clearly manifests during coordinated patrolling and vigilance among OMU leader males against all‐male band males (Xiang et al. 2014), analogous to chimpanzee boundary patrols (Wilson and Wrangham 2003) and the coordinated group defense found in several other primate species (Nunn 2000). Such cooperative male behaviors have been shown to increase male tenure lengths, resulting in access to increased numbers of adult females and offspring production in several species of mammals, birds, and fishes (Dal Pesco et al. 2022). Importantly, our results here suggest that ‘tolerance’ among OMU leader males in the *R. roxellana* multilevel society extends beyond cooperative defense against all‐male band usurpations to include ‘allowing’ the adult females of an OMU to produce extra‐pair paternity offspring, at least those offspring fathered by other OMU leader males. Individual OMU leader males thus benefit by fathering offspring with females in other OMUs.

In some other primate multilevel societies, ‘leader males’ who associated with ‘follower males’ have longer tenures, more females in their units and father more offspring than males that do not have ‘followers’ (Chowdhury et al. 2015; Snyder‐Mackler, Alberts, and Bergman 2012). Male–male bonds may enable earlier and prolonged access to reproductively active females, thus increasing tenure length and lifetime reproductive success (Dal Pesco et al. 2022). Such bonds may also function as a ‘fall‐back’ option for male primates that have lost their OMU leader status by providing support to younger, related males who still have direct access to adult females (Andersson 2017; Dickinson and Hatchwell 2004). Further research is thus required in *R. roxellana* , to determine if males deposed from former high‐ranking OMU leader status form cooperative alliances with related males that currently hold high rank within a breeding band.

### 4.4 Extra‐Pair Paternities in *R. roxellana* Reflect Female Choice

Our finding that over 50% of offspring were sired by males other than the mother's OMU leader male aligns with previous research in *R. roxellana* (Guo et al. 2010; Zhang et al. 2023). High rates of extra‐pair paternity may result from either adaptive or non‐adaptive female behaviors (Brouwer and Griffith 2019). In *R. roxellana* , females initiate mating by prostrating and presenting the rear end, curling the chest, lowering the head and giving ‘kuku’ vocalizations (Li and Zhao 2007), with extra‐pair matings previously described as covert or ‘sneaky’ (Qi et al. 2020). These behavioral patterns strongly suggest that extra‐pair mating is actively initiated by females. High frequencies of extra‐pair paternity imply that females selectively mate with males from other OMUs and with all‐male band males, some of whom later achieve OMU leader status. If extra‐pair matings imposed high costs on OMU leaders, we would expect more mate‐guarding or contest behavior. However, leader males rarely show aggression over females, with most conflicts relating to food (Guo et al. 2020), indicating that such costs may be low. We propose that cooperation between OMU leader males operates at two levels: (i) joint defense against all‐male band intrusions, and (ii) reciprocal tolerance of high rates of extra‐pair paternity within the breeding band.

Females may gain both indirect genetic benefits—such as increased immunocompetence or genetic diversity in offspring (Foerster et al. 2003; Milinski 2006; Zhang et al. 2023)—and direct benefits such as reduced infanticide risk via paternity confusion (Hrdy 1979; Palombit 2012). That many extra‐pair offspring are fathered by all‐male band males is consistent with this interpretation (Qi et al. 2020). Extra‐pair paternity rates also increased with the number of OMUs in the breeding band, suggesting that a larger pool of adult males provides females with increased mate choice opportunities. This supports the view that females actively and adaptively choose extra‐pair mates when conditions allow.

Importantly, extra‐pair paternity rates were unrelated to OMU leader male rank, contrary to our second prediction. However, many extra‐pair offspring were sired by all‐male band males who later became OMU leaders, often following female‐supported takeovers (Fang et al. 2018). This highlights the active role females may play not only in mate selection but also in male rank transitions.

Leader male tenure length was negatively associated with variation in paternity rates within OMUs, that is, the number of offspring produced within an OMU fathered by different OMU leader males. As tenure increases, males may build stronger bonds with OMU females and provide more stable access to resources (Guo et al. 2020). The benefits to females from extra‐pair mating may thus decline, while their ‘favored’ male remains in tenure, even as his reproductive performance eventually declines with age (Milich et al. 2020).

Moreover, frequent female transfers between OMUs (Yang et al. 2022) also shape mating dynamics. Transfers typically occur during the spring when new offspring are born, with females often transferring to relatively new OMUs led by leader males younger than their previous leader males. Given that most extra‐pair offspring are fathered by leader males from other OMUs, this behavior may function to further create paternity confusion and reduce infanticide risk after transfer (Van Schaik et al. 2000).

## 5 Conclusion

The mismatch between social mating systems and genetic mating systems often highlights the sexual conflict arising from the different reproductive strategies of males and females. Studies should not attempt to identify who ‘wins’ such conflicts, but to understand how differing male and female reproductive strategies both reflect and shape social and mating systems.

Although male cooperative reproductive behavior was previously thought to be rare and confined to related individuals (Van Hooff and Van Schaik 1994), male–male bonds can also arise on the basis of direct mutual net benefits (Clutton‐Brock 2002, 2009; Young et al. 2014). Here, although we found significant reproductive skew among OMU leader males, the significantly higher proportion of extra‐pair paternities that could be attributed to OMU leader males rather than all‐male band males is indicative of tolerance to other OMU males; OMU leader males benefit by producing offspring with the females within their own OMUs and in other OMUs. Coalitions of unrelated males sharing mating opportunities have not been previously reported in other primate species in which females do not disperse between groups (Toyoda et al. 2022).

Increased understanding of primate multi‐male alliances is important because similar processes may have influenced human social evolution (Dyble et al. 2015; Macfarlan et al. 2014; Rodseth 2012). Cooperation among OMU leader males in the breeding band may have allowed individual males to breed with a larger number of females and to more effectively prevent all‐male band males from accessing females. Alliance formation may facilitate coverage of more females as mating partners and increase males the likelihood of detection of rival males approaching females. That the extra‐unit paternities of leader males were unaffected by rank may therefore be due to such cooperation between OMU leader males in the breeding band. Multilevel societies allow individuals to cooperate with others beyond the individuals in their core social units. In these societies, individuals can selectively interact with specific partners from different social levels in order to cooperatively perform distinct tasks. Additionally, social units at the same level can merge to form higher‐level associations, enabling individuals to benefit from large social units without always maintaining a large core social unit (Camerlenghi and Papageorgiou 2024).

The patterns of both reproductive competition and cooperation among OMU leader males we observed, alongside active female reproductive strategies, to a limited extent mirror some key dynamics hypothesized for the evolution of multilevel societies in general. In particular, our results suggest that under certain social conditions, male–male tolerance and female‐driven mating flexibility can co‐exist and even help stabilize group structures. Such dynamics may reflect a broader evolutionary pathway whereby sexual conflict and cooperation shape group living, alliance formation, and ultimately the emergence of cohesive, multilayered societies.

## Author Contributions

**Fan Wu:** conceptualization (lead), data curation (lead), formal analysis (lead), writing – original draft (lead), writing – review and editing (lead). **Jia Liu:** formal analysis (equal), methodology (equal). **Derek W. Dunn:** formal analysis (lead), methodology (lead), writing – review and editing (lead). **Yixin Shang:** data curation (equal), formal analysis (equal). **Shiyu Jin:** data curation (equal), writing – review and editing (equal). **Huihui Du:** data curation (equal). **Yuanchun Wu:** data curation (equal). **Yiyi Men:** data curation (equal). **Guoliang Chen:** data curation (equal). **Gang He:** data curation (equal). **Baoguo Li:** conceptualization (supporting), funding acquisition (supporting), project administration (supporting), resources (supporting), supervision (supporting). **Songtao Guo:** conceptualization (lead), funding acquisition (lead), project administration (lead), resources (lead), supervision (lead), writing – review and editing (supporting).

## Conflicts of Interest

The authors declare no conflicts of interest.

## Supporting information


Appendix S1

## Data Availability

The data that support the findings of this study have been made openly available in Dryad (https://doi.org/10.5061/dryad.cfxpnvxht).
