# Supplementary material for: Beyond the Alpha: Extra‐Pair Paternities and Male Reproductive Success in a Primate Multilevel Society
Source: Ecol Evol. 2025 Jul 7;15(7):e71749. doi: 10.1002/ece3.71749 (PMC12234392; doi:10.1002/ece3.71749)
Supplement: Supplementary file 1 — Appendix S1 [file ECE3-15-e71749-s001.pdf]

Supplemental Information for:

Beyond the Alpha: Extra-pair Paternity and Reproductive Success in a Multilevel Primate Society

Fan Wu, Jia Liu, Derek Dunn, Yixin Shang, Shiyu Jin, Yiyi Men, Huihui Du,

Yuanchun Wu, Baoguo Li, Songtao Guo

Table S1. Microsatellite marker profiles and paternity analysis indices.

Locus	Primer (5′-3′)	T_A	Size	Fluo	Motif	*N*	*k*	H_O	H_E	PIC	*P-val*	PE-1	PE-2
D10s1432	Fwd:CAGTGGACACTAAACACAATCC<br>Rev:TAGATTATCTAAATGGTGGATTTCC	54	150	Fam	TATC	693	9	0.619	0.543	0.485	NS	0.156	0.3
D10s2483	Fwd:CAGGTTTTGCAATTGCTTTC<br>Rev:AACCATCTCCACCCACATAA	55	170	Fam	GATA	717	7	0.675	0.634	0.562	NS	0.205	0.352
D9s252	Fwd:ACCATGATTTGTCAACTCCTA<br>Rev:ACAATGAACATCCATATACCC	56	220	Tamra	GATA	672	6	0.637	0.544	0.496	NS	0.155	0.31
D7s2204	Fwd:TCATGACAAAACAGAAATTAAGTG<br>Rev:AGTAAATGGAATTGCTTGTTACC	54	260	Hex	TATC	609	7	0.739	0.681	0.641	NS	0.277	0.454
D6s493	Fwd:ATCCCAACTCTTAAATGGGC<br>Rev:TTCCATGGCAGAAATTGTTT	53	250	Hex	TATC	649	6	0.838	0.794	0.764	NS	0.420	0.599
D7s820	Fwd:ATGTTGGTCAGGCTGACTATG<br>Rev:GATTCCACATTTATCCTCATTGAC	61	250	Hex	GATA	676	8	0.769	0.758	0.718	NS	0.355	0.533
D19s1034	Fwd:AGGCTGTGGTGAGCTATGAC<br>Rev:GTGTCCCTAGCACCTAGCAA	60	230	Hex	GATA	644	7	0.665	0.607	0.536	NS	0.193	0.336
D19s248	Fwd:GTCCAAGGAGACAGAGCCA<br>Rev:ACTGTGCCTGACTTCTGCT	61	104	Hex	GATA	684	9	0.722	0.637	0.568	NS	0.208	0.357
D14s306	Fwd:AAAGCTACATCCAAATTAGGTAGG<br>Rev:TGACAAAGAAACTAAAATGTCCC	52	190	Tamra	GATA	614	7	0.764	0.661	0.602	NS	0.237	0.398
D6s501	Fwd:GCTGGAAACTGATAAGGGCT<br>Rev:GCCACCCTGGCTAAGTTACT	58	160	Fam	TATC	724	8	0.686	0.598	0.515	NS	0.186	0.314
D21s2054	Fwd:GCAGTAAATGTCTATGAAACAAGG<br>Rev:ATGATAGGTAGATGGATCAATTAGA	53	180	Tamra	TATC	713	8	0.691	0.541	0.435	NS	0.147	0.240

D8s1049	Fwd:TAAGTCAAACAAGCAAAGTGC<br>Rev:CCTCCTATTGCTTTTTCCAAA	55	140	Fam	GATA	694	8	0.582	0.551	0.450	NS	0.154	0.256
D10s676	Fwd:GAGAACAGACCCCCAAATCT<br>Rev:ATTTCAGTTTTACTATGTGCATGC	61	210	Tamra	GATA	680	7	0.466	0.429	0.361	NS	0.093	0.196
D12s375	Fwd:TTGTTGAGGGTCTTTCTCCA<br>Rev:TCTTCTTATTTGGAAAAGTAACCC	60	180	Tamra	TATC	589	4	0.705	0.505	0.390	NS	0.127	0.203
D7s1804	Fwd:TTCAAGTGGTTGGGTTCACT<br>Rev:TGGGTCTAGTCCAGTGGTGT	60	240	Hex	TATC	629	8	0.420	0.447	0.427	NS	0.112	0.271
D19s582	Fwd:TGTGAGCAGAGAGATGGACA<br>Rev:ACAGTGAGTTTGATCTCTAGCA	61	130	Fam	TATC	750	7	0.572	0.523	0.488	NS	0.149	0.312
D3s1766	Fwd:ACCACATGAGCCAATTCTGT<br>Rev:ACCCAATTATGGTGTTGTTACC	60	230	Hex	TATC	598	8	0.761	0.626	0.563	NS	0.215	0.368
D6s1040	Fwd:GAATGCAGGACTGTTTCTGG<br>Rev:TATATTTCTTGGGAAAGATAGATGG	60	220	Tamra	TATC	407	8	0.673	0.708	0.658	NS	0.298	0.468
D18s1371	Fwd: CTCTCTTCATCCACCATTGG<br>Rev: GCTGTCAGAGACCTGTGTTG	58	120	Fam	TATC	584	7	0.683	0.605	0.544	NS	0.198	0.349

Table S2. 2001-2015 socio-demographic OMU parameters. OMU: one-male unit.

Year	Rank	Leader Male	N females	Year	Rank	Leader Male	N females
2001	1	u	2	2006	1	c	5
	2	aj	4		2	b	6
	3	ag	3		3	a	5
	4	ai	4		4	h	5
	5	ae	4		5	d	6
	6	z	4		6	ae	5
	7	ak	2		7	e	4
	8	c	3	2007	1	b	5
2002	1	u	2		2	a	5
	2	ag	3		3	c	3
	3	ae	5		4	d	4
	4	ai	5		5	h	4
	5	z	5		6	e	4
	6	c	3		7	q	3
	7	h	4		8	ae	3
	8	am	3		9	f	1
2003	1	ag	3		10	u	2
	2	c	3		11	an	3
	3	ae	5	2008	1	a	5
	4	h	5		2	d	4
	5	b	8		3	b	3
	6	a	2		4	c	2
	7	q	4		5	e	4
	8	d	0		6	ae	2
2004	1	b	9		7	u	0
	2	c	4	2009	1	a	6
	3	ag	3		2	b	3
	4	ae	4		3	c	3
	5	a	3		4	d	3
	6	h	6		5	q	5
	7	z	3		6	e	3
	8	d	3		7	ae	3
2005	1	b	9	2010	1	a	6
	2	a	4		2	q	6
	3	c	6		3	f	6
	4	ae	6		4	e	4
	5	h	7		5	c	5
	6	d	5		6	d	4
	7	q	4		7	n	3
	8	e	2		8	u	2

2011	1	e	5
	2	f	6
	3	k	5
	4	a	7
	5	u	4
	6	v	4
	7	n	4
	8	s	5
	9	j	5
	10	ac	5
2012	1	f	9
	2	k	3
	3	e	3
	4	ah	6
	5	af	4
	6	a	2
	7	d	7
	8	v	4
	9	n	4
	10	ab	3
	11	i	2
	12	al	2
	13	c	3
2013	1	ah	6
	2	d	5
	3	k	4
	4	af	4
	5	n	4
	6	i	5
	7	a	2
	8	e	2
2013	9	v	4
	10	r	3
	11	w	2
	12	o	4
	13	g	2
2014	1	af	4
	2	i	5
	3	v	2
	4	o	4
	5	n	4
	6	w	5
	7	k	3
	8	m	4
	9	g	2
	10	p	6
	11	x	2
	12	l	1
	13	aa	3
	14	ad	2
2015	1	af	5
	2	n	3
	3	g	7
	4	i	4
	5	w	5
	6	o	4
	7	p	6
	8	v	3
	9	x	3
	10	l	4
	11	y	2
	12	t	4

Table S3. Paternity identification results. OMU: one-male unit.

Year	Offspring ID	OMU	Sire	Year	Offspring ID	OMU	Sire
2001	1	ag	d	2007	42	h	d
2001	2	e	d	2007	43	e	e
2002	3	c	a	2007	44	x	q
2002	4	c	b	2007	45	k	v
2002	5	ae	d	2008	46	a	a
2003	6	a	a	2008	47	s	a
2003	7	b	b	2008	48	s	b
2003	8	c	c	2008	49	d	d
2003	9	h	h	2008	50	s	d
2004	10	ae	a	2008	51	e	j
2004	11	c	a	2008	52	k	k
2004	12	b	b	2008	53	e	n
2004	13	c	b	2009	54	e	a
2004	14	c	b	2009	55	af	ad
2004	15	c	c	2009	56	j	b
2004	16	h	h	2009	57	c	c
2005	17	b	a	2009	58	c	c
2005	18	b	b	2009	59	e	c
2005	19	b	b	2009	60	d	d
2005	20	b	b	2009	62	e	e
2005	21	c	c	2009	63	f	f
2005	22	c	c	2009	64	b	h
2005	23	d	d	2009	65	af	j
2005	24	h	h	2009	66	s	s
2005	25	b	-	2010	66	a	a
2006	26	a	a	2010	67	b	a
2006	27	a	ae	2010	68	b	b
2006	28	b	b	2010	69	e	b
2006	29	b	b	2010	70	e	-
2006	30	e	b	2010	71	g	c
2006	31	a	c	2010	72	f	f
2006	32	c	c	2010	73	e	i
2006	33	d	d	2010	74	k	k
2006	34	e	e	2010	75	g	p
2006	35	c	q	2010	76	f	r
2007	36	a	a	2010	77	s	s
2007	37	n	a	2011	78	ad	a
2007	38	c	c	2011	79	ah	a
2007	39	d	c	2011	80	e	a
2007	40	b	d	2011	81	k	ac
2007	41	d	d	2011	82	m	b

Year	Offspring ID	OMU	Sire	Year	Offspring ID	OMU	Sire
2011	83	ac	c	2013	106	l	i
2011	84	d	c	2013	107	f	l
2011	85	e	e	2013	108	l	m
2011	86	e	e	2013	109	l	t
2011	87	f	f	2013	110	n	z
2011	88	f	f	2014	111	aa	-
2011	89	af	j	2014	112	ao	a
2011	90	k	k	2014	113	k	g
2011	91	e	u	2014	114	ao	i
2011	92	o	x	2014	115	p	l
2012	93	af	-	2014	116	af	m
2012	94	ah	-	2014	117	y	n
2012	95	d	a	2014	118	m	o
2012	96	v	g	2014	119	aa	t
2012	97	e	o	2014	120	i	d
2012	98	k	-	2015	121	g	g
2012	99	k	r	2015	122	p	i
2013	100	a	a	2015	123	w	j
2013	101	a	-	2015	124	n	l
2013	102	n	a	2015	125	aa	m
2013	103	aa	aa	2015	126	l	w
2013	104	m	ab	2015	127	g	y
2013	105	i	g	-	-	-	-

Table S4. Additional results from the statistical model 1 used to test Prediction one, in which the response variable is the number of within-unit offspring fathered by each OMU leader male in each year of the study. The summaries are given for both the full model and additional model. The results of a comparison between null model and full model and the results of likelihood ratio test for significance of each explanatory term are given (Likelihood ratio test results not shown for intercept and variables included in an interaction because these have a very limited interpretation). OMU: one-male unit.

The number of within-unit offspring fathered by each OMU leader male in each year					
**Source**	**Estimate**	**s.e.**	**Z**	***P***	
		Full model			
Intercept	-2.257	0.627	-3.595	<0.001	
Tenure	0.093	0.163	0.568	0.570	
Yearly rank	2.047	0.953	2.148	0.032	
Tenure × Yearly rank	-0.167	0.225	-0.742	0.458	
		Additional model			
Intercept	-1.947	0.436	-4.469	<0.001	
Tenure	-0.020	0.071	-0.279	0.780	
Yearly rank	1.452	0.516	2.812	0.005	
		Null model v full model			
	**df**	**AIC**	**Deviance**	**χ^2 (df = 3)**	**P**
Null	4	174.61	166.61		

Full		7	172.80	158.80	7.814	0.050

Likelihood ratio tests for significance of each explanatory term

**Model**	**Variables**	**df**	**AIC**	χ^2	**P**
Additional	Tenure	1	169.41	0.079	0.778
model	Yearly rank	1	176.53	7.200	0.007
Full model	Tenure : Yearly rank	1	171.34	0.536	0.439

Table S5. Additional results from the statistical model 2 used to test Prediction one, in which the response variable is the number of extra-unit offspring fathered by each OMU leader male in each year of the study. The summaries are given for both the full model and additional model. The results of a comparison between null model and full model and the results of likelihood ratio test for significance of each explanatory term are given (Likelihood ratio test results not shown for intercept and variables included in an interaction because these have a very limited interpretation). OMU: one-male unit.

**The number of extra-unit offspring fathered by each OMU leader male in each year**					
**Source**	**Estimate**	**s.e.**	**Z**	***P***	
		Full model			
Intercept	-1.755	0.491	-3.573	<0.001	
Tenure	0.062	0.109	0.563	0.573	
Yearly Rank	0.059	0.845	0.069	0.945	
Tenure × Yearly rank	0.057	0.188	0.303	0.762	
		Additional model			
Intercept	-1.846	0.395	-4.670	<0.001	
Tenure	0.090	0.052	1.739	0.082	
Yearly rank	0.267	0.492	0.542	0.588	
		Null model v full model			
	**df**	**AIC**	**Deviance**	**χ^2 (df = 3)**	**P**
Null	4	155.37	147.37		

Full		7	157.38	143.38	3.986	0.263

Likelihood ratio tests for significance of each explanatory term

**Model**	**Variables**	**df**	**AIC**	**χ^2**	**P**
Additional	Tenure	1	156.42	2.948	0.086
model	Yearly rank	1	153.77	0.294	0.588
Full model	Tenure : Yearly rank	1	154.34	0.009	0.763

Table S6. Additional results from the statistical model 3 used to test Prediction one, in which the response variable is the total number of within-unit paternity offspring fathered by each OMU leader male over the entire study period. The summaries are given for both the full model and additional model. The results of a comparison between additional model and full model and the results of likelihood ratio test for significance of each explanatory term are given (Likelihood ratio test results not shown for intercept and variables included in an interaction because these have a very limited interpretation). OMU: one-male unit.

**The total number of within-unit paternity offspring fathered by each OMU leader male over the entire study period**					
**Source**	**Estimate**	**s.e.**	**Z**	***P***	
		Full model			
Intercept	-2.369	0.957	-2.475	0.013	
Tenure	0.121	0.219	0.550	0.582	
Average rank	1.564	1.673	0.935	0.350	
Tenure × Average rank	0.291	0.350	0.833	0.405	
		Additional model			
Intercept	-2.978	0.731	-4.075	<0.001	
Tenure	0.298	0.062	4.818	<0.001	
Average rank	2.658	1.122	2.369	0.018	
		Additional model v full model			
	**df**	**AIC**	**Deviance**	**χ^2 (df = 1)**	**P**

Additional	4	89.198	81.198		
Full	5	90.478	80.478	0.720	0.396

Likelihood ratio tests for significance of each explanatory term

**Model**	**Variables**	**df**	**AIC**	χ^2	**P**
Additional	Tenure	1	105.588	18.390	<0.001
model	Average rank	1	92.673	5.475	<0.001
Full model	Tenure : Average rank	1	89.198	0.720	0.396

Table S7. Additional results from the statistical model 4 used to test Prediction one, in which the response variable is the total number of extra-unit paternity offspring fathered by each OMU leader male over the entire study period. The summaries are given for both the full model and additional model. The results of a comparison between additional model and full model and the results of likelihood ratio test for significance of each explanatory term are given (Likelihood ratio test results not shown for intercept and variables included in an interaction because these have a very limited interpretation). OMU: one-male unit.

The total number of extra-unit paternity offspring fathered by each OMU leader male over the entire study period					
**Source**	**Estimate**	**s.e.**	**Z**	***P***	
		Full model			
Intercept	-0.582	0.610	-0.954	0.340	
Tenure	-0.225	0.207	-1.085	0.278	
Average rank	-1.581	1.278	-1.237	0.216	
Tenure × Average rank	0.815	0.313	2.608	0.009	
		Additional model			
Intercept	-1.633	0.545	-2.996	0.003	
Tenure	0.282	0.075	3.752	<0.001	
Average rank	0.382	1.163	0.329	0.742	
		Additional model v full model			
	**df**	**AIC**	**Deviance**	**χ^2 (df = 1)**	**P**

Additional	4	98.067	90.067		
Full	5	94.568	84.568	5.499	0.019

Likelihood ratio tests for significance of each explanatory term

**Model**	**Variables**	**df**	**AIC**	**χ^2**	**P**
Additional	Tenure	1	108.972	12.905	<0.001
model	Average rank	1	96.173	0.106	0.744
Full model	Tenure : Average rank	1	98.067	5.499	0.019

Table S8. Additional results from the statistical model 5 used to test Prediction two and four, in which the response variable is the number of extra-pair paternity offspring within each OMU for each year of the study. The summaries are given for both the full model and additional model. The results of a comparison between null model and full model and the results of likelihood ratio test for significance of each explanatory term are given (Likelihood ratio test results not shown for intercept and variables included in an interaction because these have a very limited interpretation). OMU: one-male unit.

The number of extra-pair paternity offspring within each OMU for each year of the study				
**Source**	**Estimate**	**s.e.**	**Z**	***P***
		Full model		
Intercept	-0.887	0.614	-1.445	0.149
Tenure	-0.014	0.120	-0.118	0.906
Yearly rank	0.452	0.567	0.797	0.426
N_{OMUs}	0.096	0.043	2.226	0.026
Tenure × Yearly rank	-0.123	0.181	-0.682	0.496
		Additional model		
Intercept	-0.660	0.521	-1.268	0.205
Tenure	-0.090	0.052	-1.718	0.086
Yearly rank	0.149	0.353	0.423	0.672
N_{OMUs}	0.089	0.043	2.097	0.036
		Null model v full model		

	df	AIC	Deviance	χ^2 (df = 4)	P
Null	4	193.16	185.16		
Full	8	193.79	177.79	7.378	0.117

Likelihood ratio tests for significance of each explanatory term

Model	Variables	df	AIC	χ^2	P
Additional model	Tenure	1	193.34	3.091	0.079
	Yearly rank	1	190.43	0.178	0.673
	N_{OMUs}	1	194.15	3.893	0.048
Full model	N_{OMUs}	1	196.09	4.300	0.038
	Tenure : Yearly rank	1	192.25	0.467	0.495
